# EGFR endocytosis down-regulates binding to EphA2 at the plasma membrane

**DOI:** 10.1016/j.jbc.2026.113301

**Published:** 2026-06-27

**Authors:** Jennifer A. Rybak, Francisco N. Barrera

**Affiliations:** 1 Genome Sciences and Technology Graduate Program, University of Tennessee, Knoxville, Tennessee, USA; 2 Department of Biochemistry & Cellular and Molecular Biology, University of Tennessee, Knoxville, Tennessee, USA

**Keywords:** receptor tyrosine kinase, hetero-interaction- EphA2, EGFR, endocytosis

## Abstract

The epidermal growth factor receptor (EGFR) is a membrane protein that is essential to growth, differentiation, and survival in healthy cells. Misregulation of EGFR causes these pathways to function abnormally, leading to tumorigenesis. There are several therapeutics targeting EGFR at the clinic. Unfortunately, EGFR-driven tumors often develop resistance to therapeutics, hampering long-term cancer treatment with EGFR inhibitors. Overexpression of the receptor EphA2 is a common mechanism of EGFR therapeutic resistance. Importantly, EGFR and EphA2 interact at the plasma membrane, but the factors that control this interaction are poorly understood. Here, we investigate how EGFR and EphA2 interact functionally and physically. Immunofluorescence and proximity ligation data indicate that EGFR-EphA2 interactions rapidly decrease after EGFR activation with EGF. Inhibition of endocytosis blocked the effect of EGF on co-localization between both receptors, indicating that endocytosis of EGFR impairs its ability to form hetero-complexes with EphA2. Pharmacological inhibition of EGFR with tyrosine kinase inhibitors was only partially able to block this effect. Our data indicate that endocytosis is an important negative regulatory factor that impacts the levels of EGFR-EphA2 complexes. This information has implications for EGFR drug resistance in cancer.

Receptor tyrosine kinases (RTKs) are a large family of single-pass membrane proteins that recognize extracellular signals, such as growth factors, to then activate intracellular kinases and send signaling cascades through the cell. In healthy cells, these signaling cues regulate cell growth, migration, and proliferation to assist in necessary tissue functions. However, when these signals go awry, RTKs can cause tumorigenesis and metastasis. RTKs are therefore commonly targeted with anti-cancer therapeutics (1). In the classical mechanism of RTK activation, ligand binding promotes homodimerization (2). The kinase domain of the RTK homodimer then causes autophosphorylation of the receptor, which recruits adaptor proteins that trigger the phosphorylation cascades responsible for signaling (3, 4). However, it is becoming increasingly evident that homodimerization is not sufficient to fully explain the complexities of RTK function. An area of growing interest is the functional impact of hetero-interactions with other receptors. RTK heteromers have been implicated of the development of resistance to therapeutics including tyrosine kinase inhibitors (TKI). Additionally, irreproducibility between different cell systems is not uncommon when studying RTK function. Different levels of RTK hetero-interactions could potentially be behind these discrepancies, since RTK signaling cross-talk and hetero-interactions can tune signaling (5). Characterizing the RTK interactome is therefore vital to understand this important family of proteins.

The epidermal growth factor receptor (EGFR) is one of the most well-studied RTKs, and a common target for anti-cancer therapies. There are multiple generations of EGFR-targeting TKIs currently on the market and in clinical trials for cancers of the breast, colon, head-and-neck, kidney, ovary, and non–small-cell lung (5, 6, 7, 8, 9). EGFR functions through a classical mechanism where unbound EGFR is primarily monomeric, but ligand binding promotes EGFR homodimerization, cross-phosphorylation of key tyrosine residues, and receptor activation (10, 11, 12, 13). Activated receptor is then rapidly endocytosed and degraded (13, 14). EGFR is a particularly important system for the study of RTK hetero-oligomers. EGFR interacts with other members within the ErbB subfamily. Recent work has moved towards understanding cross-family interactions as well (15, 16, 17). One cross-family interaction that is notable is that between EGFR and EphA2 (18, 19).

EphA2 is a member of the Eph receptors, the largest family of RTKs (20). Like for EGFR, there are multiple EphA2-targeting drugs in clinical use, and EphA2 is implicated in a variety of diseases including glioblastoma, melanoma, and breast and lung cancer (21). However, EphA2 signals in a more nuanced way. In the presence of EphA2's ligand EphrinA1 (EA1), EphA2 forms clusters, key tyrosine residues are auto-phosphorylated, and the complex sends signals that reduce tumorigenesis (20, 21, 22, 23). Activation *via* EA1 also leads to EphA2 endocytosis and degradation (24). Alternatively, EphA2 signals without the need for ligands when kinases phosphorylate EphA2 at serine residues including S897 (25). Then EphA2 promotes signaling pathways associated with tumorigenesis (22). EphA2’s dual mode of function requires a deeper understanding of the reasons that EphA2 switches between tumorigenic and anti-tumorigenic signals, and EphA2’s interaction with EGFR is possibly one regulator of this switch.

Co-expression of EGFR and EphA2 leads to more aggressive tumors and increased drug resistance (26, 27, 28, 29). Additionally, EphA2 overexpression is a common compensatory mechanism for acquired resistance in tumors that no longer respond to EGFR-targeting TKIs (27, 28, 29, 30, 31, 32, 33). Co-inhibition of EGFR and EphA2 can resensitize otherwise resistant cells, recovering the ability of these molecules to kill cancer cells (26). Multiple studies have proposed that this compensatory mechanism is due to the direct interaction of EGFR and EphA2 (26, 28, 30).

Recent work to understand the EGFR-EphA2 interaction mechanism has focused primarily on biophysical studies that require transient overexpression of these proteins. Overexpressed EGFR and EphA2 co-immunoprecipitate (co-IP), and co-IP increases after treatment with EGFR’s ligand EGF (27). Constitutive activating mutations of EGFR and phosphomimetic mutants that simulate phosphorylation of EphA2’s key residue serine 897 (S897) each increase co-IP (27). Additionally, pulsed interleaved excitation-fluorescence cross-correlation spectroscopy experiments that track co-diffusion of fluorescently tagged EGFR and EphA2 in transiently transfected cells indicate that EGF but not EA1 promote EGFR-EphA2 binding (19). Increases in membrane concentrations of the negatively-charged lipid PIP_2_ also promote EGFR–EphA2 and EphA2–EphA2 interactions (19, 34), indicating that EGFR-EphA2 hetero-oligomerization can be regulated by membrane lipids (19, 35). While these studies make major headway in understanding the biophysical mechanisms that govern EGFR-EphA2 interactions, exogenously overexpressed proteins do not always function similarly to endogenous proteins.

Here, we investigated endogenous EGFR–EphA2 interaction in model lung cancer cell lines. We used a combination of methods that investigate EGFR and EphA2 activation, proximity, and cellular localization. We found that EGF-mediated activation of EGFR, while promoting EphA2 S897 phosphorylation, caused an overall decrease in EGFR-EphA2 proximity. This proximity decrease is correlated with EGF-induced endocytosis of EGFR. We inhibited endocytosis and then kinase signaling to find that EGF-induced decreases in EGFR-EphA2 proximity are dependent on EGFR endocytosis but not kinase signaling. Together, these data show that EGFR endocytosis is a regulator of EGFR-EphA2 interactions.

## Results

### EGFR activation promotes EphA2 phosphorylation at S897

**Figure 1:**
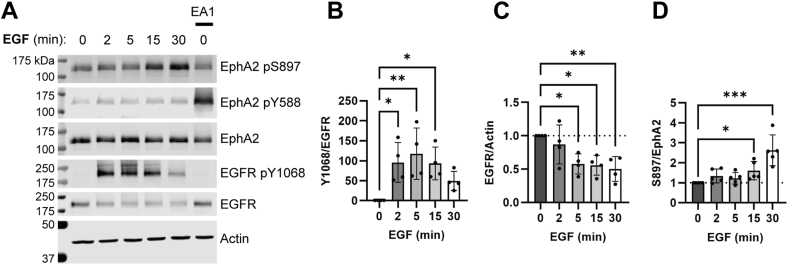
**EGF regulates EGFR and EphA2 signaling.***A*, Western blots of EGFR, EphA2, and key phosphorylation sites. We performed a time course of EGF activation. EphrinA1-Fc (EA1) treatment is showed as a control. *B*, quantification of EGFR pY1068 normalized to total EGFR levels, relative to control (0 min). N = 4. Statistical analysis was performed using a Kruskal Wallis test (H(5) = 12.62, *p* = 0.0133) with a Dunn’s test for multiple comparisons. *C*, quantification of total EGFR. N = 4. Statistics were Kruskal Wallis test (H(5) = 10.65, *p* = 0.0308) with an Uncorrected Dunn’s test for multiple comparisons. *D*, EphA2 pS897 levels normalized to total EphA2. N = 5. Kruskal–Wallis test (H(5) = 16.62, *p* = 0.0023) with an Uncorrected Dunn’s test for multiple comparisons. ∗, *p* ≤ 0.05; ∗∗, *p* ≤ 0.01; ∗∗∗, *p* ≤ 0.005. Experiments were performed in H358 cells.

We tested EGFR and EphA2 response to EGF in H358, cancer cells that natively express both receptors. We treated the cells with each protein’s corresponding ligand, EGF and EA1. Phosphorylation is commonly used to determine the activity status of RTKs. We ascertained activation of EGFR by western blotting for phosphorylation of tyrosine 1068 (pY1068) (Fig. 1*A*). EGF-mediated activation was successful, as shown by a significant increase in pY1068 that maxed out at 5 min and decreased by 30 min after EGF stimulation (Fig. 1*B*). EGFR phosphorylation was accompanied by a significant reduction in total EGFR protein levels (Fig. 1*C*), which can be attributed to the protein degradation that occurs after ligand activation (36).

EphA2 ligand-independent signaling was tracked by phosphorylation of EphA2 serine 897 (pS897) (37, 38), while ligand-dependent signaling was determined with phosphorylation of tyrosine 588 (pY588). As expected, EA1 treatment promoted EphA2 pY588 and did not affect pS897 (Fig. 1*A*), indicating EphA2 ligand-dependent signaling functions normally in our system. Additionally, EGF treatment promoted a 1.5-fold increase in EphA2 pS897 phosphorylation at 15 min and an almost 3-fold increase at 30 min (Fig. 1*D*). The observed delay between EGFR activation and EphA2 phosphorylation suggests that EGFR activation promotes downstream processes that facilitate EphA2 serine phosphorylation. This observation agrees with prior studies that propose that EGF-mediated activation of AKT is responsible for EphA2 S897 phosphorylation (27, 32, 39, 40).

### EGF causes a decrease in EGFR-EphA2 proximity that corresponds to EGFR endocytosis

**Figure 2:**
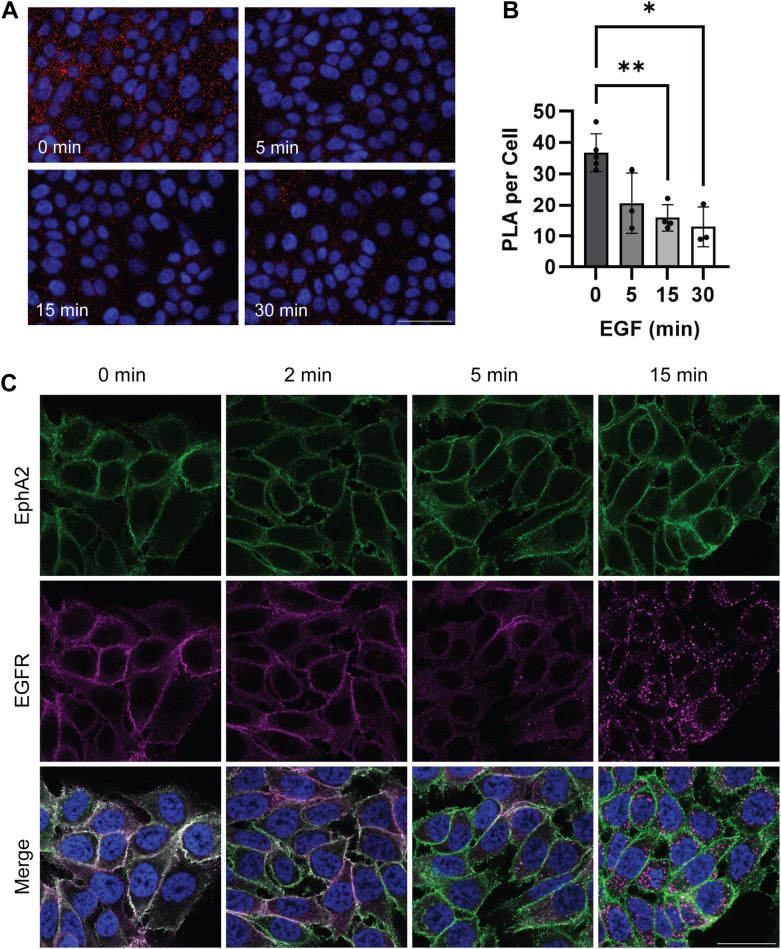
**EGF decreases EGFR-EphA2 proximity due to EGFR endocytosis.***A*, representative PLA images for proximity of EGFR-EphA2 in H358 cells in basal conditions (0 min), or after treatment with 100 ng/ml EGF for 5, 15, or 30 min. DAPI = blue, PLA = red. Scale = 50 μm. *B*, quantification of the number of PLA spots per cell. N = 4 biological replicates, n = 6 to 10 images per condition per N. Statistical analysis was performed using a Welch’s ANOVA (*W* (3,4.978) = 11.79, *p* = 0.0106) with Dunnett’s test for multiple comparisons. ∗, *p* ≤ 0.05; ∗∗, *p* ≤ 0.01. *C*, representative images of H358 cells immuno-stained for EphA2 (*green*), EGFR (*magenta*), and DAPI (*blue*) after no treatment (0 min) or EGF treatment for 2, 5, and 15 min. Scale = 30 μm.

Based on previous studies from us and others utilizing transfected proteins (19, 27), we expected that EGF-induced EphA2 S897 phosphorylation would correlate with increased EGFR-EphA2 hetero-interaction. To investigate the amount of EGFR-EphA2 interaction, we performed a proximity ligation assay (PLA) which utilizes antibodies to identify EGFR and EphA2 molecules within 40 nm, including heteromers (41). We treated the H358 cells with EGF for 5, 15, and 30 min, which includes the timepoints corresponding to maximal EGFR pY1068 and EphA2 pS897 signal. Then we fixed the cells and subjected them to PLA (Fig. 2*A*). As expected, PLA with no primary antibody showed no signal, while PLA with EGFR and EphA2 antibodies gave clear signals (Fig. S1*A*). We calculated the number of PLA spots per cell, as well as the area of the PLA signals per cell. Starting at 15 min, we observed a significant reduction in PLA (Fig. 2*B*), which suggests a decrease in the levels of EGFR-EphA2 interaction. The PLA area per cell reflected the same phenomenon (Fig. S1B). We additionally performed PLA after EA1 treatment for 5 and 15 min (Fig. S2*A*), and observed that the difference between basal and EA1 treatment was not significant (Fig. S2, *B* and *C*), suggesting that EA1 has no effect on EGFR-EphA2 heteromers, as observed with transfected proteins (19).

Because EGF-induced decreases in EGFR-EphA2 proximity was opposite to results observed with transfected proteins, we investigated the cellular localization of both proteins after EGF treatment using immunostaining (Fig. 2*C*). Under basal conditions (0 min), both proteins localized to the plasma membrane, as expected. After 5 min EGF treatment, EGFR was more intracellularly located, and by 15 min, EGFR distinctly formed intracellular puncta presumed to be the product of EGFR endocytosis (36, 42). In contrast, EphA2 remained localized to the plasma membrane at all time points (Fig. 2*C*). We therefore hypothesized that endocytosis prevents the EGFR-EphA2 hetero-interactions that have been previously detected (19, 43).

### Inhibition of endocytosis causes sustained EGFR activation

**Figure 3:**
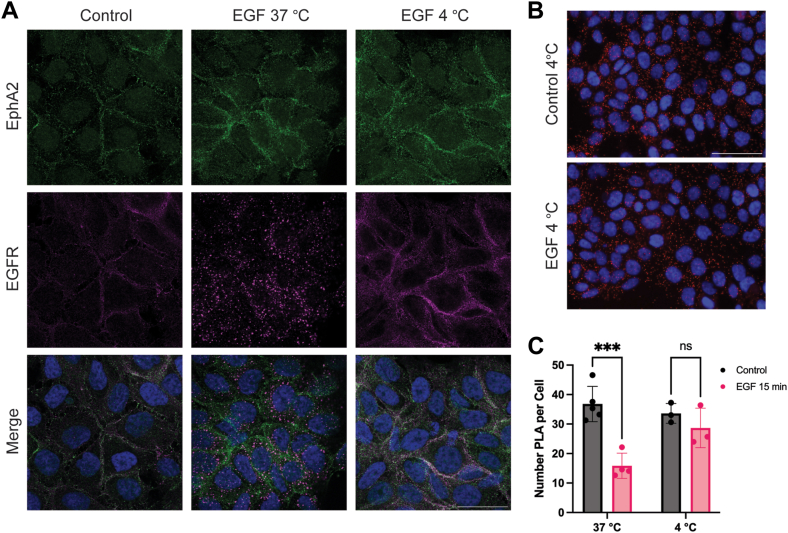
**Inhibition of EGFR endocytosis enhances EGFR-EphA2 proximity.***A*, representative images of H358 cells immuno-stained for EphA2 (*green*), EGFR (*magenta*), and DAPI (*blue*) that were treated with EGF for 15 min at 4 °C or 37 °C. Scale = 30 μm. *B*, representative images of PLA of EGFR-EphA2 in H358 cells after no treatment (Control) or EGF for 15 min at 4 °C. DAPI = *blue*, PLA = *red*. Scale = 50 μm. *C*, quantification of the number of PLA pots per cell. N = 3 to 5 biological replicates, n = 6 to 10 images per condition per N. Statistical analysis was performed using a Two-Way ANOVA where the row factor was ns and the column factor (F (1,11) = 21.22, *p* = 0.0008) with Tukey’s test for multiple comparisons. ∗∗∗, *p* ≤ 0.005.

To test how endocytosis affects EGFR and EphA2, we inhibited endocytosis prior to investigating receptor activation, proximity, and localization. To inhibit endocytosis, we modified our EGF treatment temperature from 37 °C to 4 °C. Incubation at 4°C inhibits endocytosis of EGFR by preventing receptor internalization and trafficking (44). In contrast to treatment with EGF at 37 °C, which promoted EGFR puncta formation, at 4 °C EGFR remained membrane localized after 15 min of EGF exposure (Fig. 3*A*). We repeated our PLA experiment after treatment with EGF for 15 min at 4 °C (Fig. 3*B*). The 4 °C control PLA per cell was no different than when cells were treated at 37 °C (Figs. 3*C* and S3). However, while EGF treatment at 37 °C caused a significant decrease in PLA per cell, treatment with EGF at 4 °C caused no change (Fig. 3). This result supports that endocytosis is responsible for the exhibited decreases in EGFR-EphA2 interaction.

**Figure 4:**
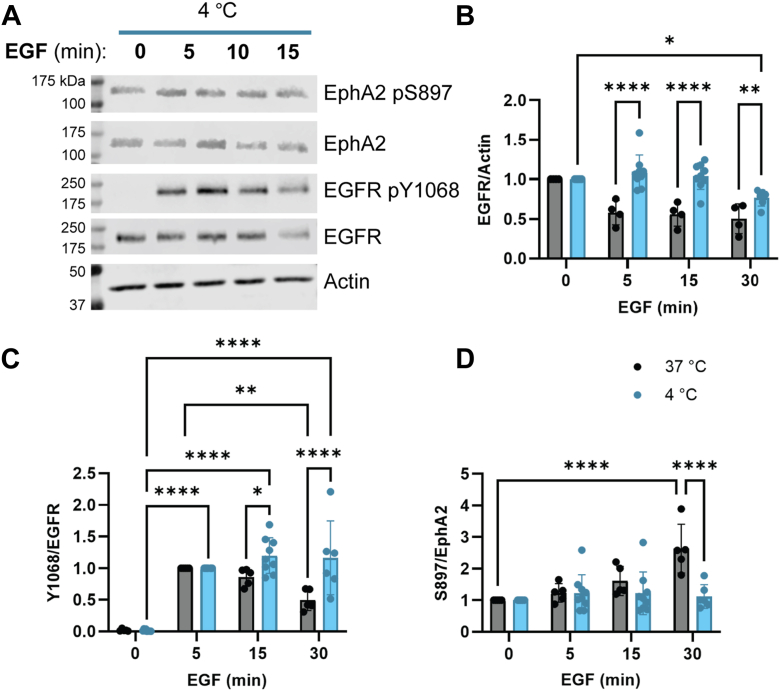
**Treatment at 4 °C preserves ligand-dependentEGFR activation, but blocks EphA2 S897 phosphorylation.***A*, Western blot analysis of EGFR, EphA2, key phosphorylation sites at 4 °C after no treatment (0) or EGF treatment for 5, 10, or 15 min. *B*, comparison of EGFR levels after EGF activation at 37 °C (*black*) and 4 °C (*blue*). *C*, phosphorylation of EGFR at Y1068. *D*, quantification of pS897 normalized to total EphA2. Statistical analysis was performed using a Two-Way ANOVA in all cases, followed by Dunnett’s test for multiple comparisons (*panel B*) or Tukey’s test for multiple comparisons (*panels C* and *D*). N = 4 to 9. ∗, *p* ≤ 0.05; ∗∗, *p* ≤ 0.01; ∗∗∗, *p* ≤ 0.005, ∗∗∗∗, *p* ≤ 0.001.

We next repeated the Western blot experiment at 4 °C (Fig. 4*A*) to rule out that the changes in EGFR-EphA2 proximity were due to misregulation of activity of the receptors. Total EGFR levels stayed consistent for 5- and 15-min EGF treatments, but at 30 min there was a small decrease (Fig. 4*B*). The EGFR levels at 30 min at reduced temperature were still larger than at 37 °C (Fig. 4*B*), indicating a delay in EGFR degradation when endocytosis is inhibited. Additionally, we observed that EGFR was properly activated at 4 °C, as shown by the effect of EGF on pY1068 EGFR levels. The decrease in EGFR phosphorylation after 30 min that occurred at 37 °C was not observed at 4 °C (Fig. 4*C*). Prolonged EGFR phosphorylation is indicative of sustained EGFR signaling. Interestingly, the EphA2 pS897 increase observed at 37 °C was not detected at 4 °C (Fig. 4*D*). This result suggests that increasing the plasma membrane levels of active EGFR is not a sufficient condition to drive EphA2 pSer signaling.

### EGFR kinase inhibition downregulates EGFR/EphA2 signaling but not endocytosis or proximity

**Figure 5:**
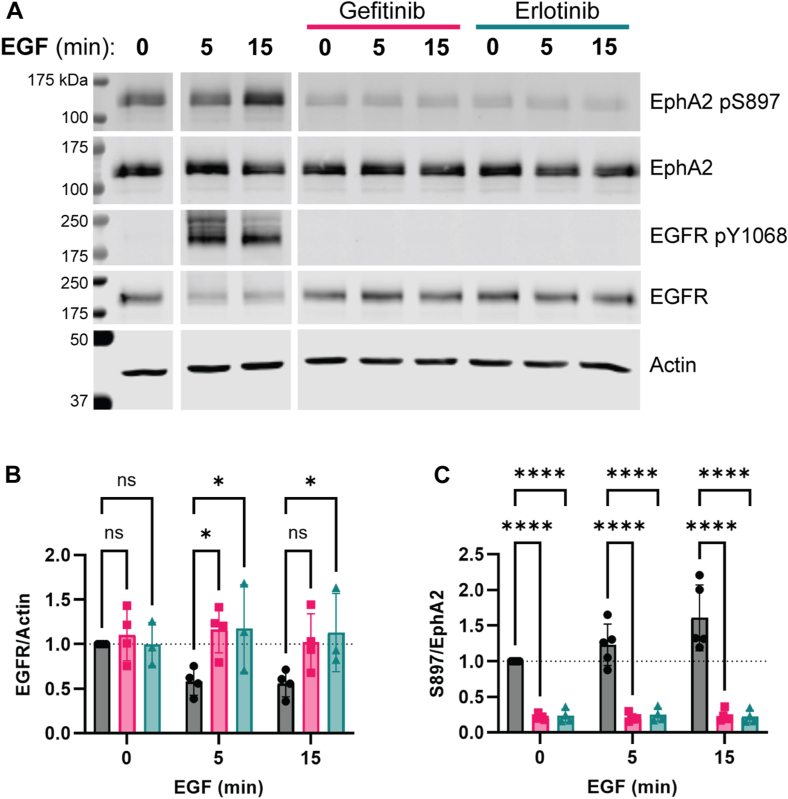
**EGFR kinase inhibitors prevent ligand-independent EphA2 activation.***A*, Western blots with the EGFR kinase inhibitors Gefitinib (Gef) or Erlotinib (Erl) for 2 h, followed by EGF treatment for 5 or 15 min, or EGF alone. The 0, 5, and 15 min lanes are also shown in Figure 1*A*. *B*, quantification of total EGFR. After actin normalization, each replicate was normalized to the control (0 min). N = 3 to 4. Statistical analysis was performed using a two-way ANOVA test where significance was only found for the column factor (F(2,24) = 7.221, *p* = 0.035) with a Dunnet’s test for multiple comparisons. *C*, quantification of EphA2 pS897, normalized to its corresponding total EphA2 band, and then each replicate was normalized to the control (0 min). N = 5. Statistical analysis was performed using a Two-Way ANOVA test (F(2,33) = 134.8, *p* < 0.0001) with an uncorrected Dunnet’s test for multiple comparisons. ∗, *p* ≤ 0.05; ∗∗∗∗, *p* ≤ 0.001.

Since we observed no decrease in EGFR-EphA2 proximity under conditions with reduced endocytosis and a lack of EphA2 pS897, we investigated whether hetero-interactions were dependent on downstream kinase signaling. We investigated the impact of inhibition of EGFR activity on the formation of the EGFR-EphA2 complex. We employed the TKIs Gefitinib (Gef) and Erlotinib (Erl), which potently inhibit EGFR kinase activity (31). Control experiments showed that, as expected, in the presence of Gef or Erl, treatment with EGF failed to induce pY1068 (Fig. 5*A*) or degrade EGFR (Fig. 5*B*), indicating that both activation and degradation of EGFR were inhibited by the TKIs. In agreement with previous reports, Gef and Erl caused a significant decrease in EphA2 pS897 from basal levels, as well as inhibited EGF-induced increases in pS897 (Fig. 5*C*) (31). Therefore, EphA2 ligand-independent signaling was reduced when TKIs inhibited EGFR. Previous studies suggest that AKT activation is responsible for EphA2 S897 phosphorylation in response to EGF (27, 31, 38, 39). To determine if AKT is responsible for the reduction of pS897 below basal levels, we additionally performed western blots for total AKT and two AKT phosphorylation sites that are indicative of activation (pS473 and pT308, Fig. S5). In all replicates, phosphorylation of AKT decreased after Gef or Erl treatment, indicating that AKT function is decreased from basal levels in the presence of the EGFR TKIs (Fig. S5). This result suggests that the EphA2 pS897 decrease is due to sustained AKT deactivation, agreeing with prior studies.

**Figure 6:**
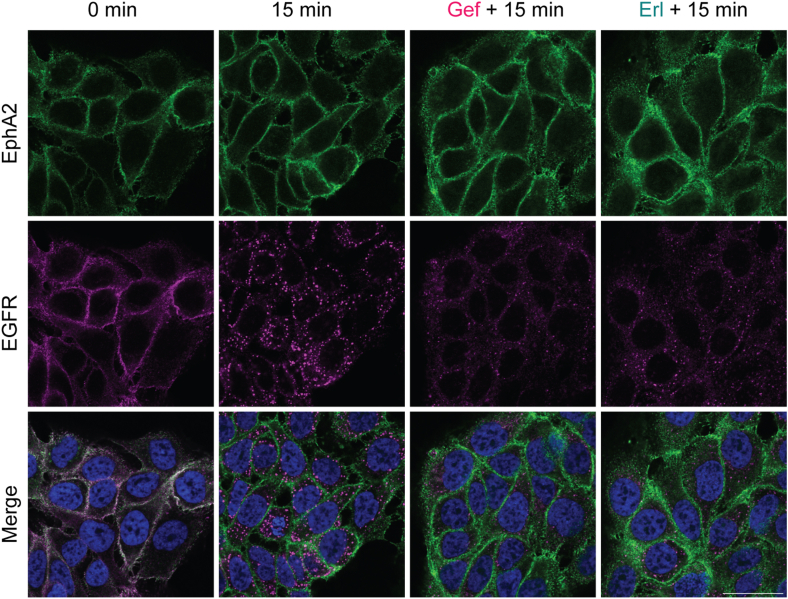
**EGFR TKIs do not completely inhibit EGFR endocytosis.** Representative images of fixed H358 cells immuno-stained for EphA2 (*green*), EGFR (*magenta*), and DAPI (*blue*). Images in the first column (0 min) show basal conditions. All other cells were treated with EGF for 15 min, or with EGF followed by kinase inhibitors (Gef + 15 min, Erl + 15 min). Scale = 30 μm. 0 min and 15 min images from Figure 2*C* are duplicated here for direct comparison of the TKI + EGF conditions.

**Figure 7:**
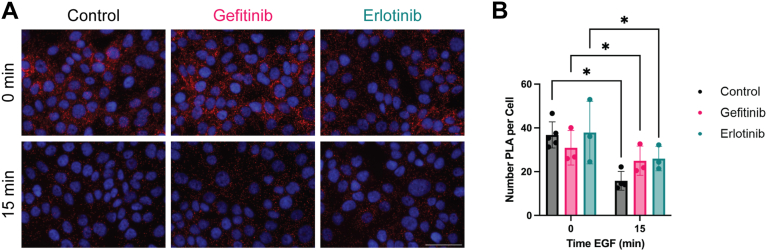
**EGF-induced EGFR-EphA2 proximity is inhibited by EGF despite EGFR kinase inhibition.***A*, representative images of PLA for EGFR-EphA2 proximity in H358 cells after treatment with kinase inhibitors Gef or Erl alone, or followed by EGF supplementation for 15 min. DAPI = *blue*, PLA = *red*. Scale = 50 μm. *B*, quantification of the number of PLA spots per cell. N = 3 biological replicates, n = 6 to 10 images per condition per N. Statistical analysis was performed using a Two-Way with Tukey’s test for multiple comparisons. ∗, *p* ≤ 0.05.

Next, we studied the effect of TKI on EGFR endocytosis. When we repeated the immunofluorescence experiments with TKI pretreatment, we observed that the samples with Gef and Erl showed EGFR internalization after EGF treatment, reminiscent of the 5 min EGF treatment in Figure 2*C*, but did not cause full puncta formation (Fig. 6). This observation was supported by quantification of these images (Fig. S6). When we performed PLA in the presence of TKI, we observed that treatment with the TKIs alone did not affect EGFR-EphA2 proximity (Fig. 7). Interestingly, when EGF was incubated after Gef or Erl treatment, the EGFR ligand still induced a decrease in PLA signal. Taken together, the TKI experiments suggest that S897 phosphorylation does not necessarily correlate with EGFR-EphA2 interactions like previously hypothesized (19, 27), suggesting that additional cellular factors are at play.

The TKI experiments indicate that the decrease in EGFR-EphA2 proximity observed after EGF treatment results from EGFR endocytosis. Additionally, neither EGFR kinase activity (pY1068), nor EphA2 ligand-independent signaling (represented by pS897) is required for EGFR endocytosis or decreased EGFR-EphA2 proximity to occur. Collectively, this suggests a decoupling of EGFR’s signaling state from the EGFR-EphA2 interaction.

## Discussion

In this study, we advance the understanding of the factors that control the formation and stability of EGFR-EphA2 complexes. Experiments in cells expressing both receptors at endogenous levels showed that activation of EGFR with the EGF ligand promotes phosphorylation of EphA2 S897 (Fig. 1), a marker of EphA2 ligand-independent tumorigenic signaling. This effect is likely due to kinases downstream of EGFR such as AKT, RSK, and ERK, which phosphorylate EphA2 S897 and subsequent oncogenic signals (18, 31). We hypothesized that there would be a direct correlate between S897 phosphorylation and EGFR-EphA2 interactions. However, PLA and confocal microscopy data indicated that this was not the case, as EGF instead led to a rapid decrease in EGFR-EphA2 interactions (Fig. 2). These results are in stark contrast with prior biophysical experiments performed using transfected EphA2 and EGFR, which showed a large increase in hetero-complex formation after EGF incubation (19, 27).

Our data indicate that the EGF-induced decrease in EGFR-EphA2 interactions are a consequence of EGFR endocytosis and degradation that follows EGFR activation by EGF (Fig. 2). Interestingly, we did not see a significant decrease in the EGFR-EphA2 interaction when we treated with EphA2’s ligand EA1, which similarly leads to EphA2 endocytosis (Fig. S2). However, the half-life of EphA2 in the plasma membrane (2.5 h) is longer than that of EGFR (1.5 h) in basal conditions, and EphA2 ligand-directed endocytosis is also slower than EGFR’s (36, 37). We therefore hypothesize that our time point of 15 min may not have been enough to observe any EA1-induced EphA2 endocytosis and degradation. However, it is also possible that EA1 did not decrease the interaction due to its previously known effects on EGFR endocytosis, as ligand-dependent EphA2 signaling inhibits EGFR recycling through inhibition of AKT (38). Future work will determine how EphA2 signaling and endocytosis directly regulate the EGFR-EphA2 interaction.

Inhibition of endocytosis *via* temperature reduction inhibited EGFR-EphA2 proximity changes (Fig. 3). However, it also inhibited EphA2 S897 phosphorylation (Fig. 4). Because EGFR TKIs (that also reduce pS897) had no effect on EGFR-EphA2 proximity (Figs. 5 and 7), we do not believe that the temperature effect on S897 was a result of differing proximity. Instead, we hypothesize that the cold temperature’s ability to hinder the movement of proteins throughout the cell is responsible for a delay in adapter protein recruitment to active EGFR followed by a subsequent delay in the downstream proteins being recruited to EphA2 for phosphorylation.

Our study also shows that EGFR TKIs cause a reduction in EphA2 pS897 regardless of EGFR’s activation state (Fig. 5). Though this mechanism is eventually overcome in EGFR TKI-resistant tumours, this function might be useful for other cases; in tumors that show increased EphA2 pS897 induced by mechanisms other than EGFR TKI resistance (39), EGFR inhibition might be a viable target. This makes the case that regardless of the original target, dual-inhibition of EGFR and EphA2 might be a viable way to reduce the development of resistance in various cancer types as well as overcome resistance once it does develop.

Interestingly, we found that complete inhibition of EGFR activation *via* TKIs was unable to completely neutralize EGFR endocytosis in response to EGF (Fig. 6). However, previous studies report that inhibition of EGFR signaling also inhibits EGFR endocytosis (45). A key difference is the EGF treatment times; Kim *et al.* reported that pretreatment with Gef inhibits endocytosis at 10 min EGF treatment while we saw incomplete inhibition of endocytosis at 15 min EGF (Fig. 7). Therefore, we believe that kinase inhibition causes a delay in EGFR endocytosis rather than a complete loss. This is supported by previous results showing that endocytosis occurs due to EGFR dimerization, not necessarily kinase function (46). Because the TKIs inhibit kinase activity but not EGFR homodimerization, endocytosis is still able to occur, albeit in a delayed fashion.

Our results generally point towards endocytosis of RTKs being a mechanism by which hetero-oligomers “turn off”, similarly to how this works for homo-oligomers. However, we cannot rule out the possibility that the EGFR-EphA2 heterodimer is additionally affecting endocytosis. Previous work with another EGFR heterodimer, EGFR-HER2, shows that HER2 negatively regulates EGFR endocytosis; specifically, EGFR and HER2 heterodimerize under basal conditions, and EGFR homodimers endocytose away from HER2, which stays at the membrane. However, when HER2 expression is reduced, EGFR endocytoses and degrades more quickly, suggesting that heterodimerization negatively regulates EGF-induced EGFR endocytosis (47). This idea is supported by the fact that EGFR-HER2 and EGFR-HER3 heterodimers do not endocytose under the same conditions that EGFR homodimers do (48). Another study shows that when EGFR-HER2 heterodimers are endocytosed, they are preferentially recycled to the membrane rather than degraded (49). Though our work shows that endocytosis negatively regulates EGFR-EphA2 heterodimers, it might also be true that the EGFR-EphA2 heterodimers negatively regulate EGFR endocytosis. More work is needed to investigate this theory.

Taken together, our data put forward the idea that endocytosis downregulates EGFR-EphA2 hetero-interactions, and generally suggests that RTK hetero-oligomers are subject to the same types of deactivation mechanisms as homo-oligomers. There are multiple avenues of investigation to further pursue. EGFR endocytoses *via* both clathrin-independent endocytosis and clathrin-mediated endocytosis depending on the concentration of EGF; at low concentrations of EGF, clathrin-mediated endocytosis prevails, and after endocytosis, EGFR is more likely to be recycled back to the membrane than degraded (50). It would perhaps be an interesting follow-up to perform a dose-response curve of EGF to determine how different endocytic pathways regulate EGFR-EphA2 interactions. We might see that recycled EGFR is more likely to bind to EphA2. Additionally, EGF is one of seven different EGFR activating ligands, and each ligand promotes varied conformational rearrangements and endocytic pathways (11). It would be interesting to determine how different EGFR ligands affect EGFR-EphA2 interactions. Finally, extrapolating our findings to other EGFR-interacting partners and the RTK interactome as a whole could be an important step to fully understand the function and regulation of RTKs.

## Experimental procedures

### Cell culture

H358 cells were obtained from ATCC and cultured at 5% CO_2_ and 37 °C in Dulbecco's Modified Eagle's Medium (DMEM) supplemented with glucose, 10% fetal bovine serum (FBS), and 100 U/ml penicillin-streptomycin. Cells were passed at 80% confluency and not cultured beyond 20 passes.

### Cell treatments

For kinase inhibitor conditions, cells were treated with the designated molecule (Gef or Erl) at 10 μM for 2 h at 37 °C prior to ligand treatment. EGF treatments were performed after inhibitor treatments, or alone, at 100 ng/ml for the designated time at 37 °C or 4 °C. EA1-Fc treatments were performed at 500 ng/ml for 5 or 15 min at 37 °C.

### Antibodies

Total EGFR (4267), EGFR pY1068 (3777), Total EphA2 (97535), EphA2 pY588 (12677), and EphA2 pS897 (6347) antibodies were obtained from Cell Signaling Technology (CST) and diluted 1:1000 for western blots. Actin (CST, 3700), anti-mouse (LiCore, 925-68020), and anti-rabbit (LiCore, 926-32211) were diluted 1:5000 for western blots. Anti-EphA2 (12927) and anti-EGFR (54359) were obtained from CST and diluted 1:100 and 1:400 respectively for immunofluorescence and PLA.

### Immunofluorescence

H358 cells were plated to 80% confluency on #1.5 12 mm sterile glass coverslips and then starved overnight in serum free media prior to treatment. Cells were then fixed in 4% paraformaldehyde in PBS for 15 min at 37 °C. Blocking and permeabilization were performed in 5% goat serum, 0.3% Triton X-100 in PBS for 1 h prior to primary incubation in blocking buffer for 2 h at room temperature. Secondary antibodies (anti-Rabbit conjugated to AlexaFluor596 and anti-Mouse conjugated to AlexaFluor488) were diluted 1:1000 in blocking buffer and incubated for 1 h at RT. Cells were then stained with DAPI at 1 μg/ml for 5 min prior to mounting the coverslips *via* Prolong Diamond mounting media overnight. Images were acquired *via* a 63× 1.4NA oil objective on a Leica SP8 White Light Laser Confocal Microscope.

### Western blot

H358 cells were plated to ∼80% confluency on 12-well plates and allowed to adhere 24 h prior to overnight starvation and then treated as described. Cells were lysed on ice for 30 min with lysis buffer (50 mM Tris-HCl, pH 7.5, 150 mM sodium chloride, 5 mM EDTA, 1% Triton X-100) containing protease and phosphatase inhibitors prior to a 10 min centrifugation at 13,000*g*. The pellet was discarded, and lysates were diluted to the same concentration. Protein was run on a 10% SDS-polyacrylamide gel and transferred onto 0.45 μm nitrocellulose. Blocking was performed with 5% milk, and primary antibodies were diluted in 5% milk or 3% BSA for total protein and phospho-protein, respectively. Primary incubation was performed overnight at 4°C. Secondary antibodies were diluted in 5% milk and incubated for 1 h. Membranes were imaged for fluorescence on an Odyssey CLx Imaging System (LI-COR). Bands were quantified using Image Studio Lite. Phospho-protein bands were normalized to the corresponding total protein levels, while total protein bands were normalized to their corresponding actin loading control bands.

### Proximity ligation assay

H358 cells were plated to ∼80 confluency on a 96-well black-sided, clear-bottom plate and allowed to adhere 24 h before overnight starvation in serum-free media. Cells were then treated as described prior to fixation in 4% paraformaldehyde for 10 min and permeabilization in 0.3% Triton X-100 for 5 min at room temperature. DuoLink Proximity Ligation was then performed *via* manufacturer’s instructions. Specifically, cells were blocked for 1 h at 37 °C in DuoLink blocking buffer and incubated with primary antibodies overnight at 4 °C diluted in DuoLink antibody diluent. DuoLink anti-Rabbit MINUS and anti-Mouse PLUS secondary antibodies were incubated for 1 h at 37 °C. Cells were then probed with DuoLink ligation buffer and ligase for 30 min prior to polymerization with DuoLink polymerase and amplification buffer for 100 min at 37 °C. DAPI staining (1 μg/ml) was performed for 5 min, and cells were left in PBS for imaging. Cells were imaged using a Cytation 5 Multi-Mode plate reader equipped with a 40× imaging objective with fluorescent channels for DAPI and RFP. The number of PLA per cell was analyzed *via* ImageJ according to previous protocols (30), where 6 to 10 images were taken per biological replicate per condition, the number of PLA per field was counted and divided by the number of DAPI-stained nuclei in the same field, and the images were averaged per replicate.

Statistical Analysis.

## Data availability

Data will be provided upon reasonable request.

## Supporting information

This article contains supporting information.

## Conflict of interest

The authors declare that they have no conflicts of interest with the contents of this article.
