# Supplementary material for: EGFR endocytosis down-regulates binding to EphA2 at the plasma membrane
Source: J Biol Chem. 2026 Jun 27;302(8):113301. doi: 10.1016/j.jbc.2026.113301 (PMC13396832; doi:10.1016/j.jbc.2026.113301)
Supplement: Supporting information [file mmc1.docx]

Supporting Information for

**EGFR endocytosis down-regulates binding to EphA2 at the plasma membrane**

*Jennifer A. Rybak and Francisco N. Barrera*

This file includes:

Figures S1 to S5


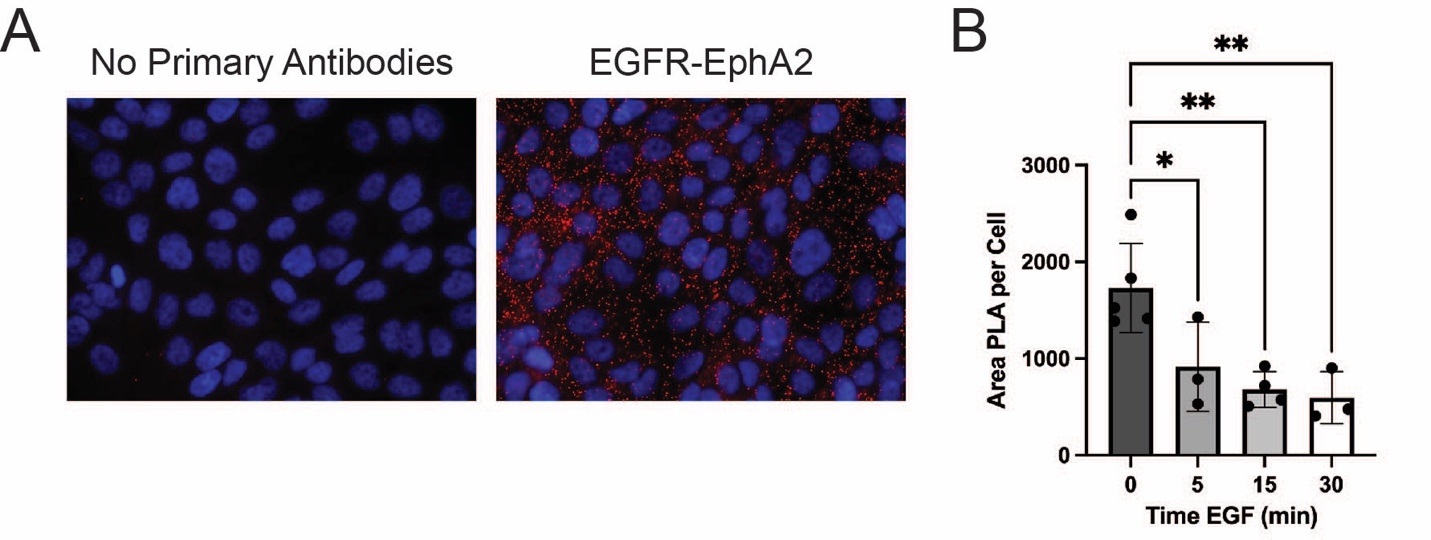


**Supplemental Figure 1: EGFR-EphA2 PLA 1**

**A.** Representative PLA images for proximity in H358 cells when no primary antibodies are used (Negative Control) or EGFR and EphA2 primary antibodies are used, showing that the lack of false positives from the PLA kit. To ease comparison, the EGFR-EphA2 image is the same as the 0 min timepoint from Figure 2A . **B.** Quantification of the area of PLA per cell from Figure 1A. N = 4 biological replicates, n = 6-10 images per condition per N, which shows similar results to the number of PLA spots per cell.


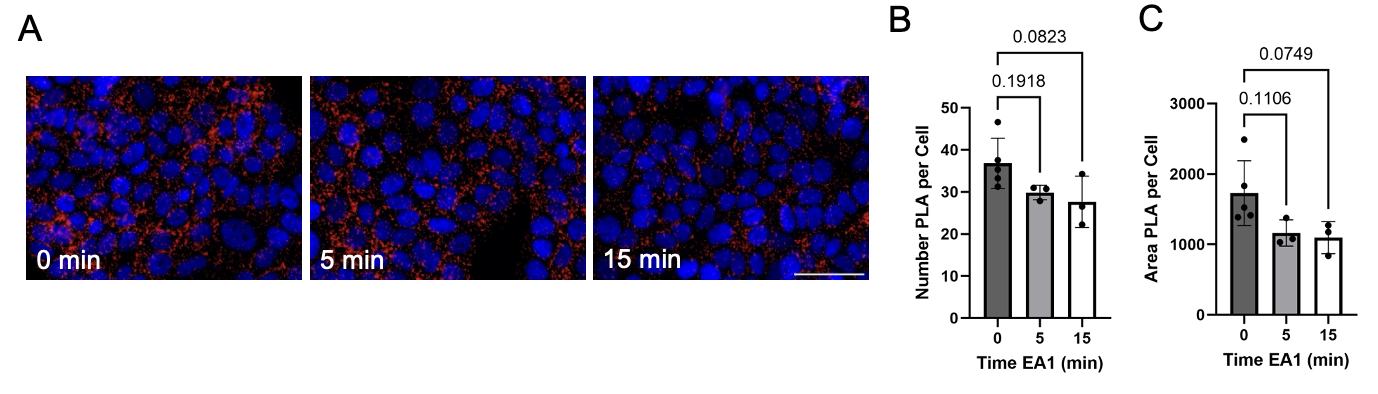


**Supplemental Figure 2: EA1 causes a trend of decreased EGFR-EphA2 PLA.**

**A.** Representative PLA images for proximity of EGFR-EphA2 in H358 cells after no treatment (0 min), or treatment with 100 ng/mL EA1-Fc for 5 or 15 min. DAPI = blue, PLA= red. Scale = 50 μm **B-C.** Quantification of the number of PLA per cell (**B**) or the area of PLA per cell (**C**) N = 3-4 biological replicates, n = 6-10 images per condition per N.


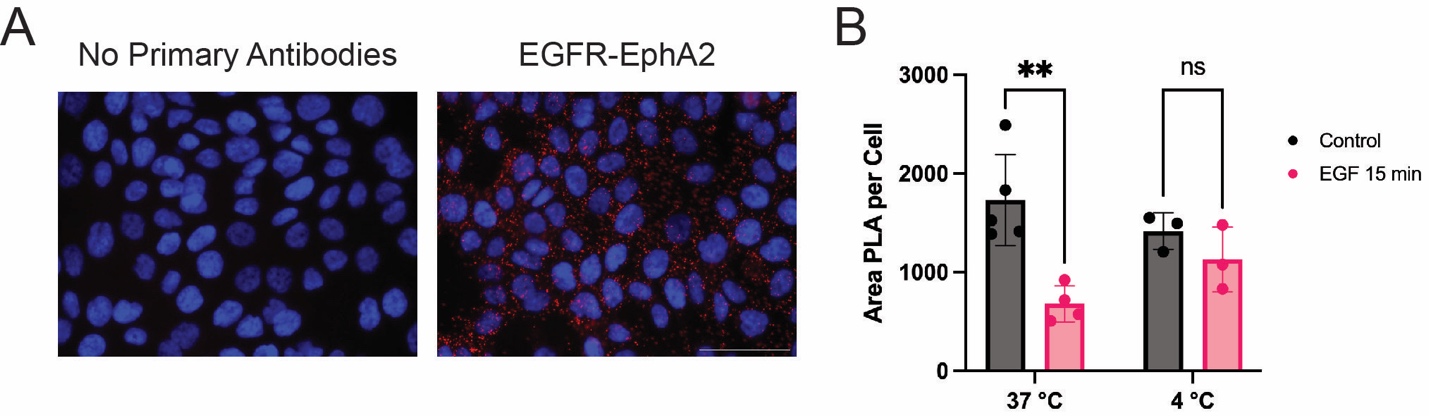


**Supplemental Figure 3: EGFR-EphA2 PLA controls for Figure 3**

**A.** Representative PLA images for proximity in H358 cells when no primary antibodies are used (negative control) or EGFR and EphA2 primary antibodies are used, showing that the lack of false positives from the PLA kit. **B.** Quantification of the area of PLA per cell from Figure 3B. N = 4 biological replicates, n = 6-10 images per condition per N. **, p ≤ 0.001.


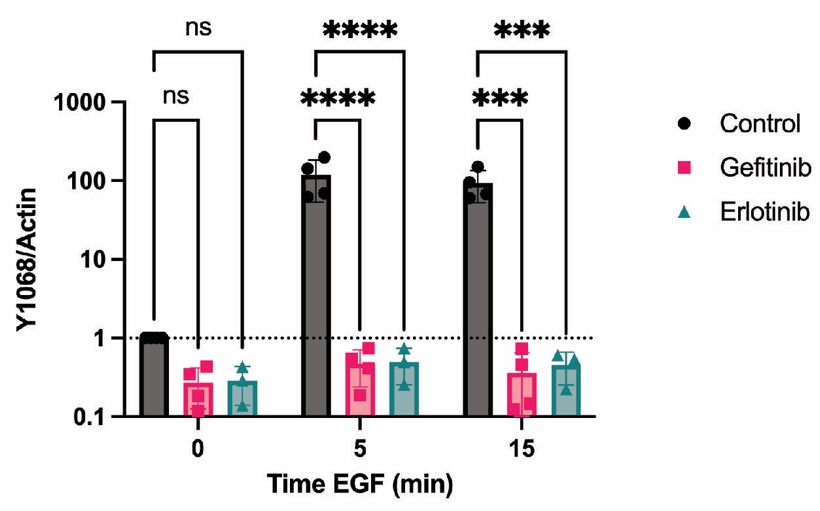


**Supplemental Figure 4: pY1068 quantification for Figure 5**

Quantification of EGFR pY1068 western blots in Figure 5, where pY1068 was normalized to its corresponding total EGFR band, and then each replicate was normalized to the control (0 min). N = 3-4.


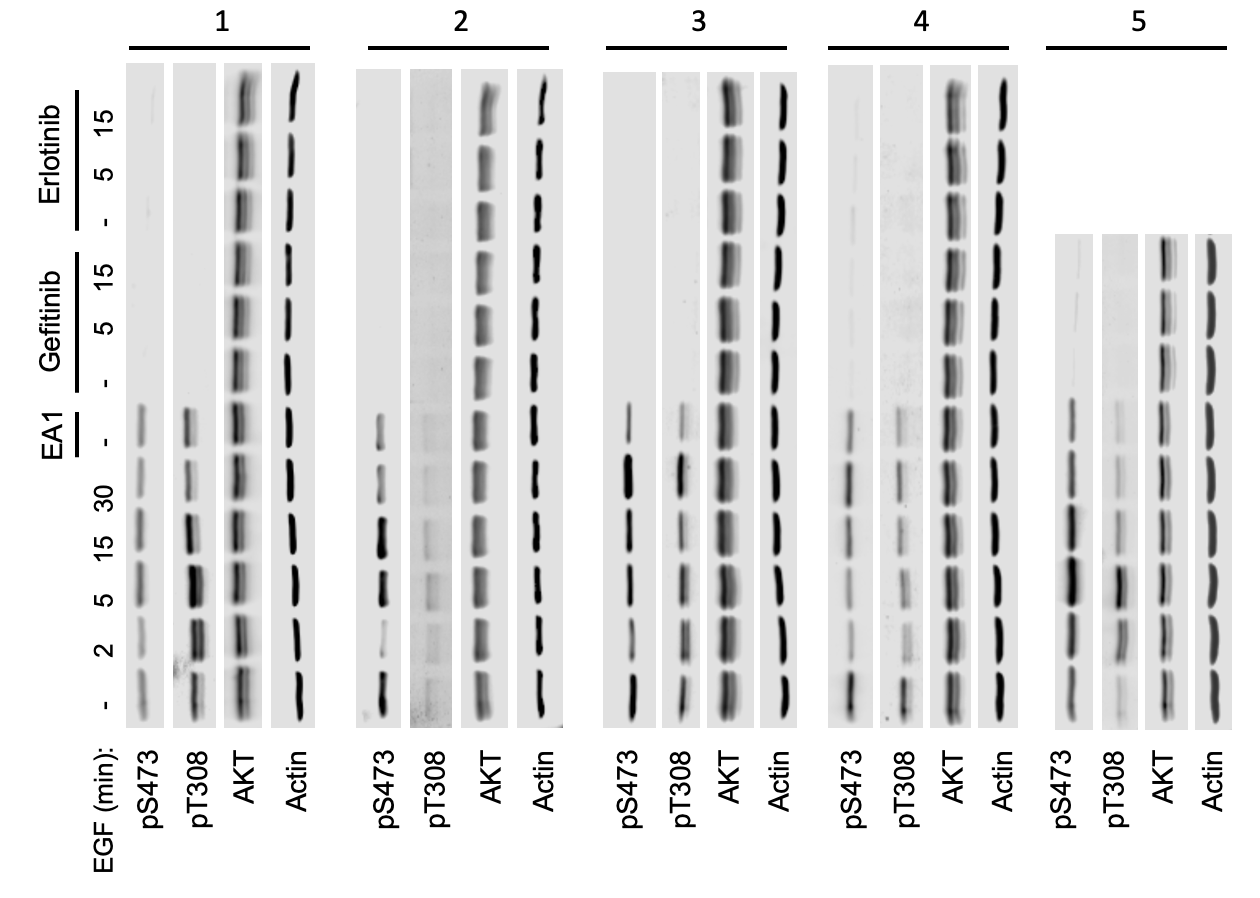


Supplemental Figure 5: EGFR TKIs inhibit AKT phosphorylation.

Western blots of total AKT, key phosphorylation sites, and actin. H358 cells were treated with EGFR kinase inhibitors Gefitinib (Gef) or Erlotinib (Erl) for 2 hr, followed by EGF treatment at 100 ng/ mL for 5 or 15 min, or with EGF alone for the designated time. Five biological replicates of the experiment are shown, with the numbers to the right indicating the replicate number. Replicate 1 was performed simultaneously to Figure 1A and Figure 5A experiments, and therefore contains the same image for the actin loading control.


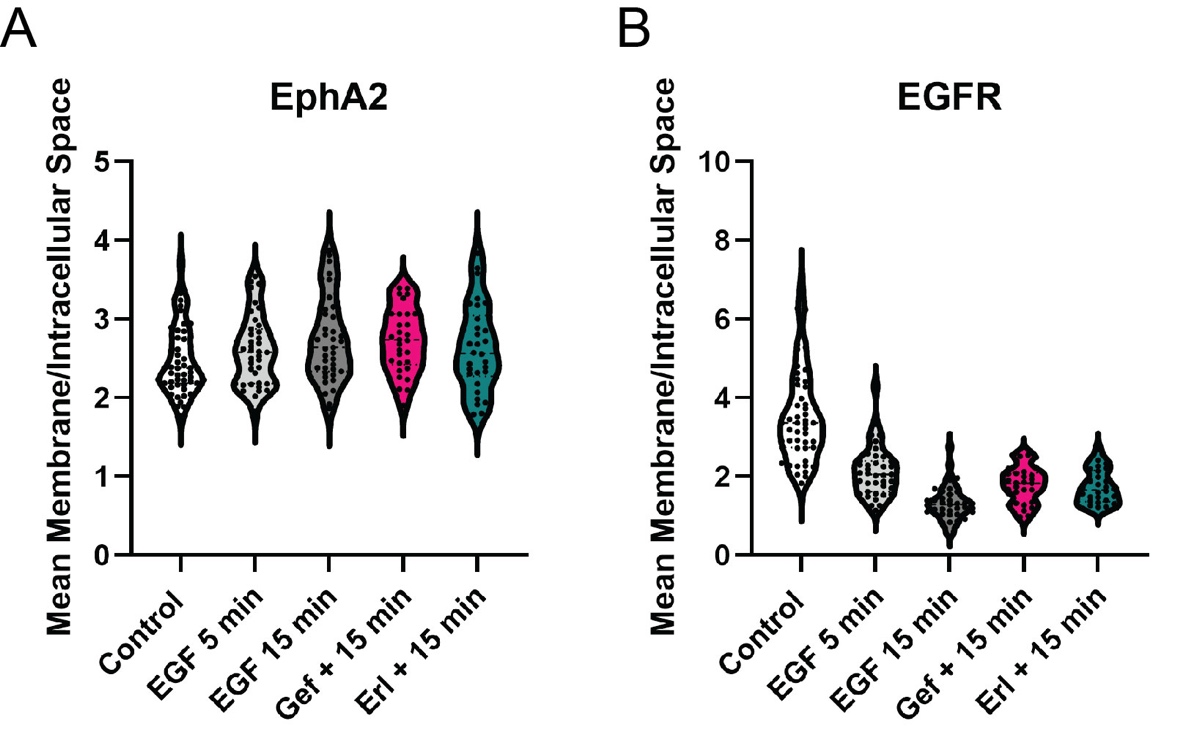


Supplemental Figure 6: Membrane quantification of IF images from Figure 6.

Mean membrane fluorescence was divided by mean intracellular fluorescence to give the membrane ratio for each channel in IF images. Each data point represents a single cell. Cells were analyzed from 2-3 independent experiments from 5-10 individual images. EphA2 shows no discernable difference in membrane localization, while EGFR shows a decrease in membrane localization over time after EGF treatment, supporting visual differences observed in the images.
